# Effect of Age and Disease Duration on the Levodopa Response in Patients with Advanced Parkinson’s Disease for Deep Brain Stimulation of the Subthalamic Nucleus

**DOI:** 10.3389/fneur.2016.00097

**Published:** 2016-06-27

**Authors:** Dursun Aygun, Ersoy Kocabicak, Mustafa Onur Yildiz, Yasin Temel

**Affiliations:** ^1^Department of Neurology, Ondokuz Mayis University, Samsun, Turkey; ^2^Department of Neurosurgery, Ondokuz Mayis University, Samsun, Turkey; ^3^Department of Neurosurgery, Maastricht Medical Center, Maastricht, Netherlands; ^4^Department of Translational Neuroscience, Maastricht Medical Center, Maastricht, Netherlands

**Keywords:** Parkinson’s disease, deep brain stimulation, levodopa response, age, disease duration, subthalamic nucleus

## Abstract

**Background:**

Deep brain stimulation (DBS) has become a preferred option for the treatment of motor symptoms in patients with advanced Parkinson’s disease (PD). A good levodopa response (LR) is considered the most important criterion in determining the suitability of a patient for DBS. However, the effect of age and disease duration (DD) on the LR is still a subject of discussion.

**Objective:**

Here, we investigated the effect of age and DD on the preoperative LR in PD patients to be selected for DBS.

**Methods:**

From August 2011 to May 2015, 54 consecutive patients (29 men and 25 women) with advanced PD were evaluated for DBS of the STN and included in this retrospective study.

**Results:**

Thirty-seven patients were found suitable for DBS of the STN and 29 of them underwent bilateral surgery. We found no significant correlation between DD and the LR. However, there was a significant negative correlation between the patients’ age and the LR.

**Conclusion:**

The results indicate that the patients’ age, rather than DD, has a negative effect on the LR. The study, therefore, indicates that PD patients with an advanced age and with a poor LR are not good candidates for DBS of the STN.

## Introduction

Parkinson’s disease (PD) is a chronic neurodegenerative disorder primarily characterized by progressive motor impairments due to dopamine insufficiency. As the disease progresses, levodopa-related motor complications develop (1). At this stage, it is difficult to treat PD patients with dopaminergic medications. Deep brain stimulation (DBS) is an effective option for the treatment of motor symptoms in patients with advanced PD (2). The success of DBS is mainly dependent on the selection of the patients suited for this treatment, who could thus have a better benefit-to-risk ratio. A good levodopa response (LR), corresponding to >30% improvement according to the Unified Parkinson’s Disease Rating Scale part (UPDRS) Part III score, is currently the most important criterion in determining the suitability of a patient for DBS (3, 4), because it is known that only PD patients who have a good LR can benefit from DBS.

To the best of our knowledge, the effect of age and disease duration (DD) on the LR in patients with advanced PD has not been investigated in detail up to now. The American Academy of Neurology has reported that DD and age have an effect on the outcome of DBS (5). Durso et al. (6) have reported that the effect of age on the LR in PD is adverse. A study of patients with early PD has pointed out that DD has a negative effect on the LR (7). Similarly, it has been suggested that the long-duration response to levodopa eventually becomes ineffective with disease progression (8). These data suggest that the LR decreases as the disease advances.

In contrast to the abovementioned studies, Ganga et al. (9) showed that the magnitude of LR did not decrease significantly in PD patients at the end of a mean follow-up of 18.2 years. Similarly, Wider et al. (10) suggested that the long-duration response to levodopa remains significant even in advanced PD. Thus, the effects of age and DD on the LR are currently debated.

Here, we report the effect of age and DD on the LR in patients with advanced PD evaluated for DBS of the subthalamic nucleus (STN). We addressed the question as to whether the preoperative LR correlates with age and DD.

## Materials and Methods

### Subjects

This retrospective study was approved by the Local Ethics Committee of our University. It was registered in the registry of our University (registration number 2015/121). From August 2011 to May 2015 (a period of 46 months), 54 consecutive patients with advanced PD (all patients except 2 had a DD longer than 5 years) evaluated for DBS were included in this study. Diagnosis of PD was based on the United Kingdom Parkinson’s Disease Society Brain Bank Clinical Diagnostic Criteria as bradykinesia in association with rest tremor, rigidity, or postural instability (11). All PD patients were assessed by a neurologist (Dursun Aygun) experienced in movement disorders. From all patients and their families, a detailed history of the demographic features of the patients and the motor and non-motor symptoms of PD, including rapid eye movement (REM) sleep behavior disorder (RBD), was obtained. Thus, DD and age for all patients were identified from this detailed disease history. Their clinical motor symptoms were assessed before DBS by the UPDRS III in both OFF (at least 12 h after the last levodopa dose) and ON medication states (12). The UPDRS III in the “ON” condition was obtained 40–60 min after administration of 1.5 times the optimal morning dose of levodopa. The LR rates of the patients were thus determined on the basis of the difference between the UPDRS III scores in the OFF and ON medication states. Both the Core Assessment Program for Surgical Interventional Therapies in PD (CAPSIT-PD) and the Florida Surgical Questionnaire for Parkinson Disease (FLASQ-PD) criteria were used to select patients suited for STN DBS (3, 13). All the patients had a history of both positive response to levodopa and a positive LR during follow-up; however, we only applied DBS surgery to those whose LR was over 30%. All data of the PD patients were recorded and saved on an electronic file.

We applied the following inclusion criteria: (1) clinical diagnosis of idiopathic PD (11) and (2) adult age (older than 18 years) and the following exclusion criteria: (1) patients whose history of DD and age were unclear, (2) patients who were not evaluated for STN DBS, and (3) patients whose initial symptoms could not be determined from their history.

The patients were classified as belonging to the tremor-dominant (TD) or non-tremor-dominant (NTD) PD subtypes. When tremors were seen at the disease onset as the sole initial symptom, the patients were included in the TD subtype (14, 15). The patients with TD had, as expected, a higher tremor score than the scores for bradykinesia and rigidity of UPDRS in the OFF medication state (9). If tremor and bradykinesia/rigidity scores were equal, the clinical descriptions by the patient and patient’s family were used to determine the dominant motor signs (9).

### Statistical Analysis

Data are presented as mean ± SD. The SPSS Version 15.0 was used for the statistical analysis. Because of the non-normally distributed data, differences between the groups were analyzed by non-parametric tests. The correlations between age and DD with the LR were investigated using the Spearman’s ρ correlation test. The Mann–Whitney *U* test was used to compare the LR between motor subtypes. A value of *p* < 0.05 was set as significance threshold.

## Results

All of the patients met the inclusion and exclusion criteria of the study. The DBS was applied bilaterally in 30 of 54 patients with PD (29 men and 25 women, Table 1). Seven of the patients were subjected to DBS surgery after the completion of this study. The DBS surgery was not applied to 17 patients (31.48%) who did not meet the criteria of the CAPSIT-PD. Thus, 37 patients (68.51%) were found suitable for STN DBS (Table 1). Mean age was 55.62 ± 9.4 years (a range of 34–74 years). Mean DD was 10.68 ± 5.3 years (a range of 3–30 years). All except two of our patients evaluated for DBS had a DD longer than 5 years. The LR rates ranged between 7.9 and 8.8%, with an average of 46.73 ± 20%. The number of the patients in the Hoehn and Yahr stage ≥3 in the OFF state was 45 (83.3%) (Table 1). Of the patients, 53.7% (*n* = 29) were included in the NTD subtype and 46.3% (*n* = 25) in the TD subtype (Table 1). The Hoehn and Yahr stage, DD, and age did not differ between the subtypes.

**Table 1:** **Data of the 54 cases of the present study**.

Clinical data	Quantitative data
Age: years; mean ± SD	55.62 ± 9.4
Sex: female/male; *n* (%)	25/29 (46.3/53.7)
Subtypes: TD/TND; *n* (%)	25/29 (46.3/53.7)
Disease duration: years; mean ± SD	10.68 ± 5.3
Levodopa response: total (%; mean ± SD)	46.73 ± 20.4
Levodopa response: TD/NTD (%; mean ± SD)	49.78 ± 18/44.10 ± 21^a^
HY OFF^b^: ≥ 3 *n* (%)	45 (83.3)
Suitable for *DBS of the STN n* (%)	37 (68.5)
*DBS of the STN* performed (%)	30 (55.5)
Motor improvement at the third month^c^ (%; mean ± SD)	54.4 ± 17.9

*^a^No difference between the motor subtypes in the levodopa response*.

*^b^OFF period: the period of at least 12 h after the last levodopa dose*.

*^c^Motor improvement at the third month: the response to stimulation (stim) ON-medication (med) OFF when compared with stim OFF-med OFF in 24 patients*.

*HY, Hoehn and Yahr; TD, tremor-dominant; NTD, non-tremor dominant; STN DBS, subthalamic nucleus deep brain stimulation*.

**Figure 1:**
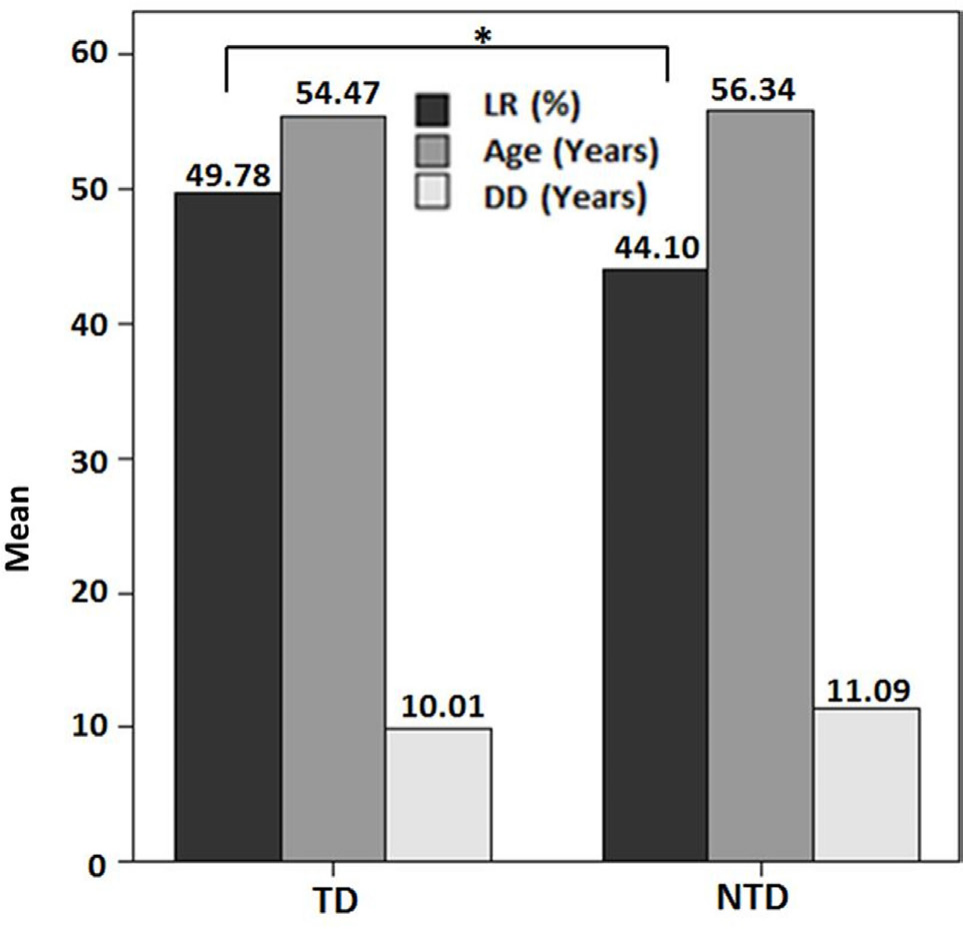
Preoperative levodopa response in the motor subtypes of Parkinson’s disease in this study (*p > 0.05). Abbreviations: DD, disease duration; LR, levodopa response; NTD, non-tremor dominant; TD, tremor dominant.

**Figure 2:**
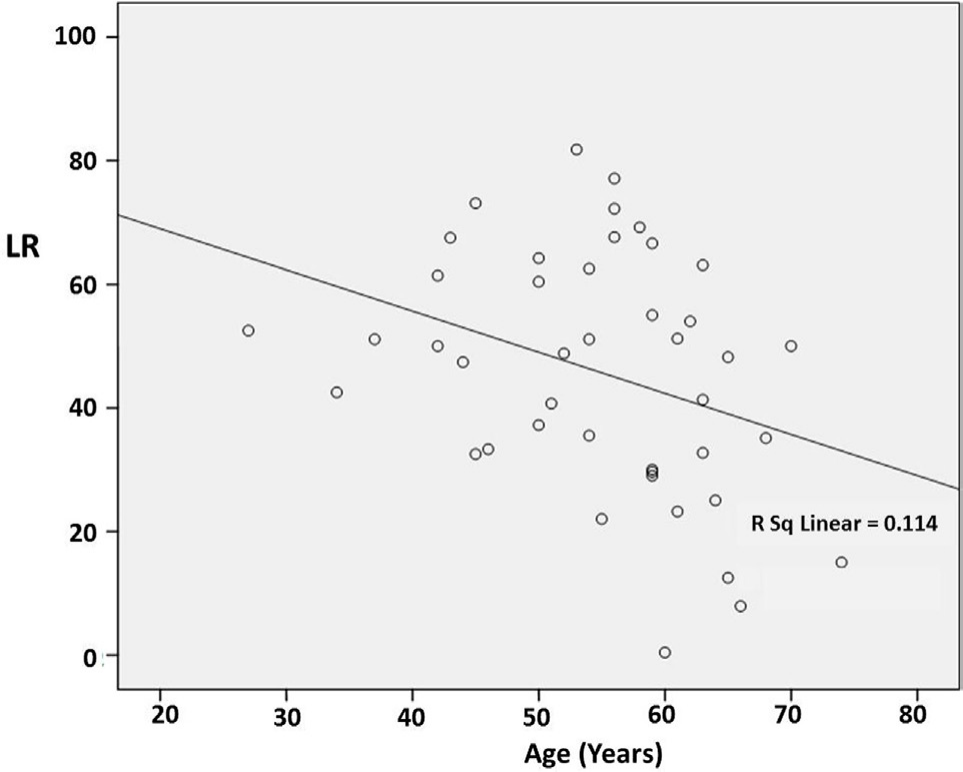
**Scatter plot of the correlation between age and the preoperative levodopa response (LR)**. The line indicates a negative correlation (*r* = −0.374; *p* = 0.013).

**Figure 3:**
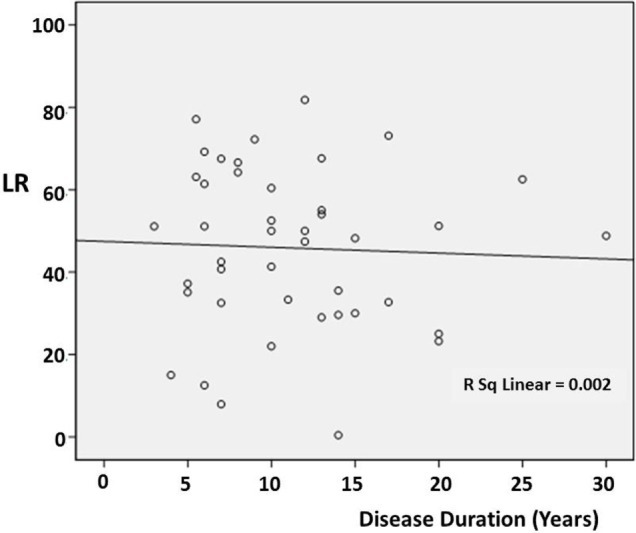
**Scatter plot of the relationship between disease duration and the preoperative levodopa response (LR) in the different cases**. The line indicates the average value.

There was no significant correlation between DD and the preoperative LR [correlation coefficient (*r*) = −0.103; *p* = 0.510] (Figure 1). However, there was a significant negative correlation between age and the preoperative LR (*r* = −0.374; *p* = 0.013) (Figure 2). There was no difference between the motor subtypes concerning the LR (Table 1; Figure 3). The effect of DBS in all patients after surgery was over 50%. For example, the motor improvement at the third month of 24 patients was 54.4 ± 17.9%, representing the response to stimulation ON/medication OFF condition when compared with stimulation OFF/medication OFF condition. There were no irreversible complications in the peri- and postoperative period of the patients who underwent STN DBS.

## Discussion

Here, we found that DD had no significant effect on the LR, while we identified a negative correlation between age and the preoperative LR. To the best of our knowledge, this is the first report on the effect of age, DD, and motor subtypes on the LR in patients with advanced PD.

Limitations of this study are the small size of the patients’ cohorts and the retrospective nature of the methodological design. However, it should be considered that all the preoperative and postoperative data of each patient have been carefully recorded by a neurologist expert in movement disorders.

Concerning the available literature, Ganga et al. (9) assessed the LR in PD during a mean 18.2-year study period (range: 14.4–21.8 years) and found that there was no significant difference in the amplitude of LR between motor subtypes (i.e., TD and TND) (9). They (9) also showed that the magnitude of LR did not decrease significantly at the end of the study period. Thus, such findings (9) support our data that motor subtypes and DD have no significant effect on the LR in PD. Another study (10) compared the postoperative and preoperative UPDRS III scores in 30 patients with STN DBS and suggested that the long-duration response to levodopa remained significant even in advanced PD, and that DBS of the STN compensated the LR (10). The results of the two abovementioned studies (9, 10) support the conclusion of our study that DD has no significant effect on the LR. Durso et al. (6) suggested that in PD patients treated long term, age has a stronger adverse influence than DD on the magnitude of the LR. Our study is, therefore, supported by such conclusions (6).

It is known that PD patients have a better response to levodopa than those with Parkinson plus syndromes (PPS) and that this poor LR in PPS is associated with pre- and postsynaptic dopamine cell degeneration. In PPS, the striatum is involved early and severely (16–18). ^11^C-raclopride positron emission tomography (PET) studies have shown that there is no decrease in binding to the postsynaptic striatal dopamine D2 receptors in patients with PD, which is increased or normal (16). This condition, considered to be a compensatory reaction to the loss of the nigrostriatal dopaminergic neurons, corresponds to postsynaptic striatal dopamine D2 receptor upregulation (16). The presence of postsynaptic degeneration (i.e., reduced striatal D2 receptor uptake or striatal hypometabolism) in PPS, in contrast to PD, has been shown in studies using striatal functional imaging, such as ^11^C-raclopride PET, ^123^I-IBZM SPECT, and FDG-PET (16–19). By means of ^18^F-DOPA, it has been shown that the loss of nigrostriatal dopaminergic terminals is more severe in PPS than in PD (20). Thus, PPS are refractory to levodopa treatment, whereas the benefit of levodopa treatment in PD can still persist in the later stages of disease due to striatal D2 receptor upregulation (16, 17). Thus, it is likely that, in PD, the LR is not significantly influenced by DD.

Taken together, these literature data support our finding that DD in PD has no significant effect on the LR. However, we have identified, in this study, a significant correlation between age and preoperative LR. The negative effect of age on the LR could be due to a slowdown in the striatal postsynaptic D2 receptor upregulation with increasing age in PD.

In contrast to the abovementioned studies, the results of other studies have pointed to the presence of prominent negative effects on the LR of DD (7, 8, 21). Espay et al. (7) have reported that DBS of the STN performed in “early” PD confers a greater quality-adjusted life expectancy than delayed surgery. It is well known that only PD patients who have a good LR can benefit from DBS. On the other hand, in patients at an earlier stage of PD, DD is obviously shorter than in those with advanced disease. Hence, it has been reported that patients with shorter DD respond better to levodopa than those with longer DD (7). These results point to an inverse correlation between DD and LR. Accordingly, it has been suggested that the long-term LR is gradually reduced with disease progression (8). A long-term follow-up study (21) has revealed a progressive worsening of UPDRS III motor score in the “stimulation OFF/medication ON” conditions over time (21). This indicated a relationship between LR and DD (21), which appears in conflict with thus study, despite the fact that the methodology for determining the LR (21) was similar to that we adopted in our study. However, we adopted a different methodology for the assessment of the relationship between the LR and DD, since, at variance with such previous investigation (21), we here compared directly these parameters. Merello et al. (22) assessed the sensitivity and specificity of an acute challenge with levodopa to predict sustained long-term levodopa responsiveness in PD. They observed that the sensitivity was lowest in patients with initial UPDRS III score ≥21 (22). These findings may imply that the sensitivity to acute levodopa challenge decreases as the disease stage advances, suggesting that the LR decreases as the disease advances.

All these sets of data could indicate that patients with a longer DD have a lower LR, than those with a shorter DD. Although we did not find a correlation between DD and LR, supporting previous studies (9, 10), our data show that DD exerts a much lower adverse influence than age on the LR, in agreement with Durso et al.’s findings (6). Such lower adverse influence of DD on LR could be associated with the progression of disease stage. However, the disease progression rate shows high interindividual variability. For example, while some PD patients rapidly develop movement difficulties, others may have mild symptoms for a relatively long time, which could be reflected in studies of patients’ populations. The effect of age and DD on the LR is an ongoing discussion topic. However, Albanese et al. (23) have reported that, with the appropriate indication and setting, acute challenge tests are useful in diagnosis and therapy of PD.

## Conclusion

In this study, we found that DD has no significant effect on the LR, and we show, instead, a significant negative correlation between age and LR, as supported by a previous study (6). Our findings suggest that patients with longer DD, but not advanced age, still benefit from DBS. Additionally, we found that there is no difference between motor subtypes concerning the LR.

## Author Contributions

EK has planned this study and has contributed in writing this article. DA has planned this study and has contributed in writing this article. MY has helped in writing and checking this article. YT has planned and looked over this article.

## Conflict of Interest Statement

The authors declare that the research was conducted in the absence of any commercial or financial relationships that could be construed as a potential conflict of interest.
